# Erythropoietin combined with traditional Chinese medicine for chemotherapy-induced anemias: A protocol of systematic review and meta-analysis

**DOI:** 10.1097/MD.0000000000022799

**Published:** 2020-10-30

**Authors:** Long-Feng Wang, Shu-Zheng Song, Jin Huang, Chuan-Hui Dou

**Affiliations:** a Department of Traditional Chinese Medicine, The Third People's Hospital of Lanzhou; b Department of Traditional Chinese Medicine, The 940^th^ Hospital of Joint Logistics Support Force of Chinese People's Liberation Army; c Department of The Second Pediatric Orthopedics, Gansu Provincial Hospital of Traditional Chinese Medicine; d Department of Hematology, Gansu Provincial Hospital of Traditional Chinese Medicine, Lanzhou, Gansu Province, China.

**Keywords:** chemotherapy-induced anemias, erythropoietin, meta-analysis, systematic review, traditional Chinese medicine

## Abstract

**Background:**

As far as we know, several systematic review and meta-analysis have assessed the safety and efficacy of erythropoiesis-stimulating agents (ESAs) in the patients with chemotherapy-induced anemia (CIA). But no study assesses the safety and efficacy of ESAs combined with traditional Chinese medicine (TCM). The aim of our study is to assess the efficacy and safety of ESAs combination with TCM for patients with CIA and will provide a higher level of evidence for clinical applications.

**Methods:**

This protocol adheres to the preferred reporting items for systematic reviews and meta-analysis protocol statement. The source of literature will be a structured search of the following 7 electronic databases: PubMed, Embase, Cochrane Library, Web of Science, China National Knowledge Infrastructure, Chinese Biomedical Literature Database, and Wanfang Database. Records will be independently evaluated by 2 reviewers. Disagreements will be resolved through consensus or third-party adjudication. Review Manager 5.3 software (Cochrane Collaboration, Copenhagen Denmark) will be used to perform meta-analysis. For dichotomous variables, odds ratio with 95% confidence intervals will be obtained by the Mantel–Haenszel method. For continuous data, mean difference with 95% confidence intervals will be used. *P* < 0.05 will be considered to be statistically significant.

**Results:**

This study will be performed to test the efficacy and safety of ESAs combined with TCM for CIA in patients with cancer.

**Conclusions:**

The result of this study will be promoted mainly in 2 ways: publish in peer-reviewed journals in the fastest way; and promotion in domestic and foreign conferences.

**INPLASY registration number:**

INPLASY202080041.

## 1 Introduction

Cancer rank second in the global cause of death and is a major health care problem that endanger the health of all mankind.^[1]^ Global cancer statistics in 2018 showed that there were 18,078,957 new cancer patients and 782,685 deaths.^[2]^ The widespread use of chemotherapy improves the survival of patients with cancer, but its adverse effects (AEs) cannot be ignored. Chemotherapy-induced anemia (CIA) is one of the AEs in patients with cancer who are treated with myelosuppressive chemotherapy and can negatively impact the patient's prognosis.^[3]^ The treatment options for CIA included iron supplementation, blood transfusion, and erythropoiesis-stimulating agents (ESAs).^[4]^ ESAs can raises hemoglobin (Hb) levels, reduces the need for red blood cell (RBC) transfusions and providing benefit to patients and healthcare systems in patients with CIA.^[5]^ However, studies have also reported that the use of ESAs in patients with cancer increased mortality during active study phase and risk of cancer progression or recurrence, and decreased survival.^[6,7]^ Therefore, the Food and Drug Administration and the European Medicines Agency added a boxed warning to the labels for ESAs at the same times.^[8]^ In 2008, the Food and Drug Administration also updated darbepoetin alfa prescribing information, decreasing the Hb treatment initiation threshold to <10 g/dL and also added limitations of use (when the anticipated outcome is cure, it is not indicated for patients receiving myelosuppressive chemotherapy).^[8]^ Despite this, there has been no better alternative in recent years. In the latest American Society of Clinical Oncology/American Society of Hematology Clinical Practice Guideline, ESAs (including biosimilars) may be offered to patients with CIA whose cancer treatment is not curative in intent and whose Hb has declined to <10 g/dL.^[9]^

However, patients with CIA had better choices in China. Traditional Chinese medicine (TCM) has very good effects in treating anemias. For example, Zhao MM et al's study showed that Danggui Buxue Decoction in combination with conventional western medicine (CWM) for renal anemia might be superior to CWM alone and there was no adverse event in the experimental group by systematic review.^[10]^ Similarly, Dang ZB et al found that TCM with the role of supporting Qi and enriching blood may be a safe and treatment for cancer related anemia.^[11]^ But as for as we know, several systematic review and meta-analysis have assessed the safety and efficacy of ESAs in the patients with CIA.^[8,12–14]^ The aim of our study is to assess the efficacy and safety of ESAs combination with TCM for patients with CIA and provide a higher level of evidence for clinical applications.

## 2 Methods

### 2.1 Study registration

This protocol adheres to the Preferred Reporting Items for Systematic Reviews and Meta-Analysis Protocol statement.^[15]^ Before that, this study has been registered in the International Platform of Registered Systematic Review and Meta-analysis Protocol (INPLASY PROTOCOL, registration number: INPLASY202080041 (10.37766/inplasy2020.8.0041)) on June 26, 2020. If this protocol needs to revise, we will also make corrections simultaneously in INPLASY PROTOCOL.

### 2.2 Ethics and dissemination

As a systematic review and meta-analysis which based on previously published literature, ethical approval and informed consent from patients are not required.^[16]^ After our study is completed, it will be published in a peer-reviewed journal.

### 2.3 Search strategy

An experienced librarian will draw up a set of search terms. The source of literature will be a structured search of the following 7 electronic databases: PubMed, Embase, Cochrane Library, Web of Science, China National Knowledge Infrastructure, Chinese Biomedical Literature Database, and Wanfang Database. Medical subject heading and text words will be searched as our keyword. Table 1 shows the search strategy of PubMed. Other database can use this search strategy after being adapted and tailored that using Boolean operators (OR/AND), truncations, proximity operators, and Medical subject heading, as appropriate for each database. Search of all database will be independently performed by 2 reviewers from inception to August 1, 2020. In search, there will be no language and publication status restrictions. In order to avoid missing potential study, we will manually search grey literature (eg, trial registries), and search for reference lists of relevant trials and reviews.

**Table 1:**
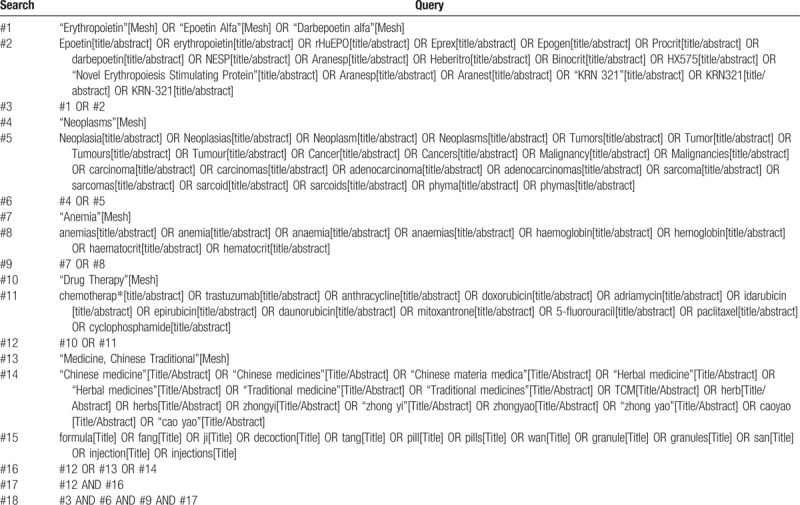
Search strategy of PubMed.

### 2.4 Eligibility criteria

The eligibility criteria for our systematic review and meta-analysis are as follows:

Participants:

(1) Patients with solid tumors confirmed by histology or cytology, do not limit the type and stage of the tumor.
(2) Patients must be at least 18 years old, and no restriction of gender, country, and race.
(3) Patients received chemotherapy, and no restriction on the type and course of drugs use.
(4) Hb ≤10.0 g/dL, and confirmed caused by chemotherapy drugs.

Interventions/comparators: Cancer patients in the control group must have received short or long acting ESAs to reduce CIAs. ESAs must be administered subcutaneously or intravenously. There is no treatment duration and dosage restrictions of ESA. Patients in the experimental group received TCM treatment on the basis of the treatment in the control group. If necessary, patients in both the control group and the experimental group were to receive RBC transfusions or other basic therapy (eg, iron). In addition, patients in both groups mush receive identical care.

Outcomes: The primary outcome is the efficacy, we expect to be evaluated from 6 aspects:

(1) the proportions of patients with an Hb increase of ≥1 g/dL;
(2) the proportions of patients with an Hb increase of ≥2 g/dL;
(3) time to first Hb increase of ≥1 g/dL;
(4) time to first Hb increase of ≥2 g/dL;
(5) the proportions of patients receiving blood transfusions, either RBCs or whole blood; and
(6) time to first transfusion.

The secondary outcome is the safety that will be assessed by the occurrence of AEs.

Study characteristics: Randomized controlled trials and controlled clinical trials will be included in this systematic and meta-analysis. Other types of studies, such as expert opinions, review papers, case reports, and case series, will be excluded.

### 2.5 Study selection

**Figure 1:**
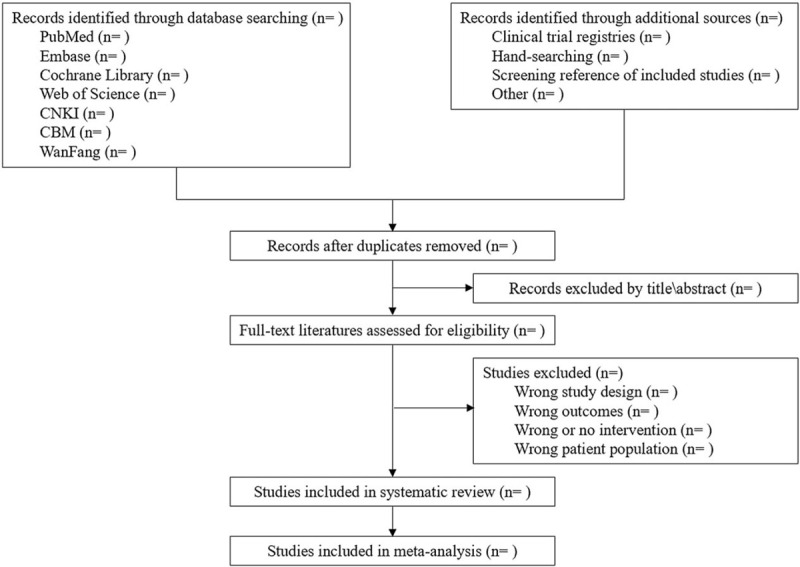
The PRISMA flow diagram of study selection process. PRISMA = preferred reporting items for systematic reviews and meta-analysis.

All records from 7 databases will be imported into EndNote X8 (Thomson Reuters [Scientific] LLC Philadelphia, PA) software to manage the identified records and remove duplicates. Next, records will be independently evaluated by 2 reviewers. First, they will be evaluated by title and abstract. Second, all potentially eligible studies will be retrieved and full-text literature reviewed to determine eligibility. Disagreements among 2 reviewers will be resolved through consensus or third-party adjudication. A Preferred Reporting Items for Systematic Reviews and Meta-Analysis flow diagram will be prepared to document the study selection process in the final publication (Fig. 1).

### 2.6 Data extraction

Same as study selection, data will be extracted independently by 2 reviewers. Disagreements will be dissolved through consensus or third-party adjudication. A standardized, predesigned, electronic data collection form implemented in Microsoft Excel 2016 will be used to extract data. Three to 5 included studies will be pre-extracted. If necessary, the forms shall be continually modified until the final data extraction form complete. The following items that included general characteristics and specific trial characteristics will be extracted: author, year of publication, country where the study was performed, funding, study duration, sequence generation, allocation sequence concealment, blinding, incomplete outcome data, and selective outcome reporting, patients (eg, total number, setting, age, sex, country, sociodemographic details, diagnostic criteria), interventions (eg, drug name, dose, and course of drugs), outcomes.

### 2.7 Missing data management

If there are missing data in several included studies, we will contact the corresponding author via email to request any insufficient or missing data. If the data is still unavailable, we will perform data synthesis from existing information and address the potential impact of missing data on the merged result in the discussion section.

### 2.8 Assessment of risk of bias

The quality of including studies will be evaluated by 2 different tools. Cochrane Risk of Bias tool will be used to assess the quality of randomized controlled trials.^[17]^ The tool for evaluating the risk of bias consists of 7 specific domains: sequence generation; allocation concealment; blinding of participants and personnel; blinding of outcome assessment; incomplete outcome data; selective outcome reporting; and other bias. Each included study will be assigned a level of risk of bias (high risk, unclear risk, low risk) for each domain. Newcastle–Ottawa scale will be used to assess the quality of case control studies.^[18]^ It is included items within 3 domains to evaluate bias in patient selection, comparability, and outcome assessments. Each included study will be awarded a maximum of 1 star for each numbered item among the items that evaluate patient selection and outcome assessments. A maximum of 2 stars will be given for comparability, and the total scores ranged from 0 to 9 points. Two reviewers will independently assess the quality for each included study, and a third reviewer will to consulate the disagreement.

### 2.9 Data synthesis

Review Manager 5.3 software (Cochrane Collaboration, Copenhagen, Denmark) will be used to perform meta-analysis. For dichotomous variables, odds ratio with 95% confidence intervals will be obtained by the Mantel–Haenszel method. For continuous data, mean difference with 95% confidence intervals will be used. The statistical heterogeneity of studies included in the meta-analysis will be assessed using *I*^*2*^ statistic (with values of 25%, 50%, and 75% is representative of the low, medium, and high heterogeneity, respectively). If the heterogeneity is high, the random effects model will be used for the analysis. And the fixed effects model will be used for studies with low or moderate heterogeneity. A sensitivity analysis will be performed by excluding 1 study at a time to test the robustness of the pooled results. If relevant data are available, subgroup analysis will be performed (eg, kinds of TCM, course of treatment, patient's gender, types of cancers). Publication bias will be evaluated using the Egger test and Begg test when at least 10 studies are included. *P* < 0.05 will be considered to be statistically significant. In addition, meta-regression will be used to determine factors that may influence the outcome. For meta-regression analyses, STATA16 (Stata Corp LP, TX) will be used.

## 3 Discussion

As a common complication of myelosuppressive cancer treatment, Moderate to severe anemia is associated with symptoms such as fatigue, dyspnea, tachycardia, and depression.^[19,20]^ This situation will negatively affect both physical functioning and quality of life of cancer patients.^[20]^ In TCM theory, CIA can be classified as the area of “XueKu,” “XuLao” according to it clinical symptoms. It can improve the symptoms of anemia in patients with cancer by strengthening the body, replenishing qi and promoting blood, reconciling the spleen and stomach, invigorating the kidney and essence, soothing the liver and blood. However, no systematic review and meta-analysis is performed to test the efficacy and safety of ESAs combined with TCM for CIA in patients with cancer. It is necessary and can provide scientific evidence for clinical and future studies. At present, we have completed the preliminary literature search, and it is estimated that this systematic review and meta-analysis can be completed in 1 year. The final result of this study will be promoted mainly in 2 ways:

(1) publish in peer-reviewed journals in the fastest way; and
(2) promotion in domestic and foreign conferences.

## Author contributions

**Data curation:** Long-Feng Wang, Chuan-Hui Dou.

**Formal analysis:** Shu-Zheng Song.

**Funding acquisition:** Jin Huang.

**Methodology:** Chuan-Hui Dou.

**Resources:** Shu-Zheng Song, Jin Huang.

**Software:** Shu-Zheng Song.

**Writing – original draft:** Long-Feng Wang, Chuan-Hui Dou.

**Writing – review & editing:** Long-Feng Wang, Chuan-Hui Dou.
